# Ultraviolet Radiation Technologies and Healthcare-Associated Infections: Standards and Metrology Needs

**DOI:** 10.6028/jres.126.014

**Published:** 2021-08-20

**Authors:** Dianne L. Poster, C. Cameron Miller, Richard A. Martinello, Norman R. Horn, Michael T. Postek, Troy E. Cowan, Yaw S. Obeng, John J. Kasianowicz

**Affiliations:** 1 National Institute of Standards and Technology, Gaithersburg, MD 20899, USA; 2 Yale School of Medicine and Yale New Haven Health, New Haven, CT 06510, USA; 3 Seal Shield, LLC, Orlando, FL 32801, USA; 4 University of South Florida, Tampa, FL 33612, USA; 5 International Ultraviolet Association, Chevy Chase, MD 20815, USA; 6 Columbia University, New York, NY 10027, USA

**Keywords:** disinfection, dose, efficacy, hospitals, innovation, light, metrology, optics, public health, standards, ultraviolet-C radiation, UV-C

## Abstract

The National Institute of Standards and Technology (NIST) hosted an international workshop on ultraviolet-C (UV-C) disinfection technologies on January 14–15, 2020, in Gaithersburg, Maryland, in collaboration with the International Ultraviolet Association (IUVA). This successful public event, as evidenced by the participation of more than 150 attendees, with 65% from the ultraviolet technology industry, was part of an ongoing collaborative effort between NIST and the IUVA and its affiliates to examine the measurement and standards needs for pathogen abatement with UV-C in the healthcare whole-room environment. Prior to and since this event, stakeholders from industry, academia, government, and public health services have been collaboratively engaged with NIST to accelerate the development and use of accurate measurements and models for UV-C disinfection technologies and facilitate technology transfer. The workshop served as an open forum to continue this discussion with a technical focus centered on the effective design, use, and implementation of UV-C technologies for the prevention and treatment of healthcare-associated infections (HAIs) in complex hospital settings. These settings include patient rooms, operating rooms, common staging areas, ventilation systems, personal protective equipment, and tools for the reprocessing and disinfecting of instruments or devices used in medical procedures, such as catheters and ventilators. The critical need for UV-C technologies for disinfection has been amplified by the outbreak of severe acute respiratory syndrome coronavirus 2 (SARS-CoV-2), the virus that causes coronavirus disease 2019 (COVID-19), stimulating an even greater emphasis on identifying testing and performance metrology needs. This paper discusses these topics based on the international workshop and community activities since the workshop, including a public World-Wide-Web–based seminar with more than 500 registered attendees on September 30, 2020; an international conference on UV-C technologies for air and surface disinfection, December 8–9, 2020; and a webinar on returning to normalcy with the use of UV-C technologies, April 27 and 29, 2021. This article also serves as an introduction to a special section of the Journal of Research of the National Institute of Standards and Technology, where full papers address recent technical, noncommercial, UV-C technology and pathogen-abatement investigations. The set of papers provides keen insights from the vantage points of medicine and industry. Recent technical developments, successes, and needs in optics and photonics, radiation physics, biological efficacy, and the needs of future markets in UV-C technologies are described to provide a concise compilation of the community’s efforts and the state of the field. Standards needs are identified and discussed throughout this special section. This article provides a summary of the essential role of standards for innovation and implementation of UV-C technology for improved patient care and public health.

Supplementary material is available at https://doi.org/10.6028/jres.126.014s.

## Table of Contents

1. Introduction……………………………………………………..………..... 3
2. Healthcare-Associated Infections ………………………………..…….......7
3. Background on UV Radiation ……………………………………..…….....9
4. NIST and Community Engagement for the Development of UV Efficacy Standards ………………………………………………………………...14
  4.1 Dialogue with Industry on NIST Capabilities at the Gaithersburg, Maryland, Campus ,,…………………………………………………14
  4.2 Engaging Stakeholders through IUVA’s 2018 Americas Conference ……………………………..……………………………………..…..15
  4.3 IUVA Healthcare Working Group ……………………………..…....16
  4.4 Workshop at Yale School of Medicine and Yale New Haven Health ……………………………..…………………………………………16
  4.5 Continued Community Engagement 2017–2020 ………………..…..17
5. NIST–IUVA Workshop on UV Technologies for Healthcare and Metrology Needs ………………………………………………………………..…...18
  5.1 NIST’s Role ……………..………………………………………..…19
  5.2 Challenges for UV Technology Development and Deployment in Healthcare ……………..………………………………………….....19
  5.3 Recommendations ………………..……………………………….....20
    5.3.1 UV-C for Health ……………………………………….….…..20
    5.3.2 UV-C for Innovation ……………………………………...…...21
    5.3.3 UV-C for Security …………………………………..................21
    5.3.4 Measures of Success …………………………………….….....22
6. Outcomes of the Workshop ……………………………………….……...22
  6.1 Recapitulation and Updates at the 2020 IUVA Americas Conference ……………………………………..………………………………...22
  6.2 Technical Perspectives from Industry via a Public Webinar, September 30, 2020 …………………………………………………...................23
  6.3 International Conference on UV Disinfection for Air and Surfaces, December 8–9, 2020 ………………………………..……………….24
  6.4 Public Webinar on “Enhancing the New Normalcy with UV Disinfection,” April 27 and 29, 2021 ……………...…………….…..24
7. Looking Ahead: Standards, Innovation Ecosystems, and Technology Transfer ………………………………………...………………….…….24
  7.1 Standards ………………………………………….…..……….…….24
  7.2 Innovation Ecosystems …………………………………..……,……25
  7.3 Technology Transfer ……………………………………...…………26
8. References …………………………………….…………………………..^28^

## 1 Introduction

This article is the preamble to a Special Section on Ultraviolet Technologies for Public Health in volume 126 of the *Journal of Research of the National Institute of Standards and Technology.* Papers in this section address recent technical, noncommercial, UV technology and pathogen-abatement investigations (Table 1). Research results in optics, photonics, radiation physics, and biological efficacy are presented. Industry successes in innovation, experimental studies, and perspectives from the field supporting the use of UV technologies for healthcare applications give a concise compilation of the current state of the field for UV-C (200 nm to 280 nm; see Sec. 3) disinfection technologies in healthcare. These articles are a valuable resource, not only for specialists in this area, but also for researchers from industries and other fields in academia or public health who are interested in this interdisciplinary field of research and development.^1^1 The authors are not presenting advocacy positions, but rather material for assessing the viability and effectiveness of approaches for UV technologies in healthcare, which may then be further considered by stakeholder groups.

**Table 1:** Papers in the Special Section on Ultraviolet Technologies for Public Health in volume 126 of the *Journal of Research of the National Institute of Standards and Technology.*

Authors	Title	Key Messages
Arthur Kreitenberg and Richard A. Martinello	Perspectives and Recommendations Regarding Standards for Ultraviolet-C Whole-Room Disinfection in Healthcare	Patient well-being must be the driving force for determining standards for disinfection systems based on ultraviolet-C (UV-C) radiation. Specific, evidence-based recommendations regarding room description, organism selection, carrier material, quantity, orientations, and locations are provided. https://doi.org/10.6028/jres.126.015
Daniel B. Spicer	Methods and Mechanisms of Photonic Disinfection	Photonic disinfection of air, surfaces, and liquids using UV-C wavelengths (200 nm to 280 nm) and blue wavelengths (400 nm to 420 nm) are discussed. These wavelengths of interest are effective disinfection tools and can augment traditional infection-prevention techniques. https://doi.org/10.6028/jres.126.016
Pawel de Sternberg Stojalowski and Jonathan Fairfoull	Comparison of Reflective Properties of Materials Exposed to Ultraviolet-C Radiation	The reflectivity of material lining the inside of a disinfection chamber can have a dramatic effect on the UV-C radiation dose received across all sides of a contaminated object. An experimental comparison of four different materials is provided to determine their efficacy as UV-C reflectors by using a custom-designed testing apparatus utilizing a UV-C radiation-emitting diode alongside photochromic UV-C indicators. https://doi.org/10.6028/jres.126.017
Ernest R. Blatchley III, Brian Petri, and Wenjun Sun	SARS-CoV-2 Ultraviolet Radiation Dose-Response Behavior	UV dose-response behavior describes the intrinsic kinetics of UV inactivation, and as such, it represents a key piece of information for the design of UV disinfection systems that are intended for inactivation of SARS-CoV-2. A summary of information that describes inactivation of SARS-CoV-2 by exposure to germicidal UV radiation is provided. https://doi.org/10.6028/jres.126.018
Stephen F. Yates, Giorgio Isella, Emir Rahislic, Spencer Barbour, and Lillian Tiznado	Effects of Ultraviolet-C Radiation Exposure on Aircraft Cabin Materials	Fabric and plastic materials commonly used in aircraft cabins were exposed to UV-C radiation to determine their sensitivity to cumulative damage from frequent application. The intensity of UV-C radiation incident on a surface depends not only on the distance between the lamp and the surface, but also on the angle between the surface and the incident beam. https://doi.org/10.6028/jres.126.019
Alisha Geldert, Halleh B. Balch, Anjali Gopal, Alison Su, Samantha M. Grist, and Amy E. Herr	Best Practices for Germicidal Ultraviolet-C Dose Measurement for N95 Respirator Decontamination	An understanding of best practices in UV-C dose measurement for N95 respirator decontamination is essential to the safety of medical professionals, researchers, and the public. The fundamental optical principles governing UV-C irradiation and detection are discussed, as well as the key metrics of UV-C wavelength and dose to inform best practices for UV-C dose measurement for N95 respirator decontamination during crisis-capacity conditions. https://doi.org/10.6028/jres.126.020
Mahsa Masjoudi, Madjid Mohseni, and James R. Bolton	Sensitivity of Bacteria, Protozoa, Viruses, and Other Microorganisms to Ultraviolet Radiation	Data concerning the sensitivity of various organisms to ultraviolet (UV) radiation exposure are very important in the design of UV disinfection equipment. This review analyzes fluence data from almost 250 studies and organizes the data into a set of recommended fluence values for specific reductions and an appendix containing all the collected data. https://doi.org/10.6028/jres.126.021
Yaw S. Obeng, Brian J. Nablo, Darwin R. Reyes, Dianne L. Poster, and Michael T. Postek	Broadband Dielectric Spectroscopy as a Potential Label-Free Method to Rapidly Verify Ultraviolet-C Radiation Disinfection	Microwave (MW) sensors offer noninvasive, real-time detection of the variations in the electromagnetic properties that characterize biological materials. The application of MW sensors using broadband MW dielectric spectroscopy coupled to a fabricated biological thin film is demonstrated for evaluating UV-C exposure effects. https://doi.org/10.6028/jres.126.022
Kumari Moothedath Chandran, Praveen C. Ramamurthy, Kawkab Kanjo, Rohan Narayan, and Raghu Menon	Efficacy of Ultraviolet-C Devices for the Disinfection of Personal Protective Equipment Fabrics and N95 Respirators	An evaluation of two UV-C disinfection devices for viricidal efficacy on personal protective equipment and N95 respirators through controlled experiments using the H1N1 virus is presented. The effectiveness of chemical disinfectants and experiments for material selection, UV dose calculation, and UV endurance of samples of personal protective equipment are also discussed to provide a systematic method to validate the efficacy of UV-C disinfection products. https://doi.org/10.6028/jres.126.023
Pablo Fredes, Ulrich Raff, Ernesto Gramsch, and Marcelo Tarkowski	Estimation of Ultraviolet-C Doses from Hg Lamps and Light-Emitting Diodes to Disinfect Surfaces	There is no generally accepted computational procedure to determine the minimum irradiation times and UV-C doses required for reliable and secure disinfection of surfaces. A mathematical model is presented to estimate irradiance distributions, and the relevant parameters are defined, including wavelength, emitted optical power, radiant efficiency, consumed power, characteristics and geometry of the irradiated surfaces, and the position of the irradiated surfaces in relation to the UV-C source. https://doi.org/10.6028/jres.126.025
Yuqin Zong, Jeff Hulett, Nomasa Koide, Yoshiki Yamaji, and C. Cameron Miller	Mean Differential Continuous Pulse Method for Accurate Measurements of Light-Emitting Diodes and Laser Diodes	A mean differential continuous pulse method for measurement of light-emitting diodes and laser diodes is presented. The method is a significant improvement relative to the differential continuous pulse method because it eliminates three major sources of error (current amplitude, pulse width variation, and pulse period variation) by measuring the mean current of the continuous pulses using a digital multimeter or a high-accuracy digitizer. (Article to be published.)
Shelby Claytor, Roger Campbell, Ashton Hattori, Eric Brown, Christopher Hollis, Max Schureck, Howard Atchley, John Stone, Michael Grady, Benjamin Yang, and T. Robert Harris	Portable Ultraviolet-C Disinfection Systems for COVID-19	The development and performance of a portable chamber for reliable UV disinfection of personal protective equipment are discussed. The design and construction of two 280 nm UV-C disinfection chambers, one using light-emitting diodes and the other using mercury vapor lamps, are presented, as well as efforts to increase the uniformity and overall intensity of UV radiation through the installation of porous polytetrafluoroethylene. (Article to be published.)
Dianne L. Poster, C. Cameron Miller, Michael A. Riley, Andras E. Vladar, John D. Wright, Christopher D. Zangmeister, Jeremy Starkweather, John Wynne, Jason Yilzarde, and Matthew Hardwick	Decontamination of N95 Filtering Facepiece Respirators using Ultraviolet Germicidal Irradiation: Assessment of Virus Inactivation, Dosage Requirement, Material Integrity, Filtration Performance, and Fit	The use of a commercial UV germicidal irradiation enclosure that delivers a substantial dose of UV-C over a short period of time is demonstrated as a useful tool for disinfecting full-face respirators. The UV-C radiation dose as a function of virus inactivation is presented, and the integrity of the respirator materials is measured via high-resolution scanning electron microscopy and tensile strength, gas flow, and particle filtration testing, and evaluation of the preservation of fit following the exposure of the respirators to UV-C radiation. (Article to be published.)
Dianne L. Poster, Michael T. Postek, Yaw S. Obeng, John J. Kasianowicz, Troy E. Cowan, Norman R. Horn, C. Cameron Miller, and Richard A. Martinello	Examining Potential Models for an Ultraviolet-C Research and Development Consortium	The development of an international precompetitive collaborative UV research consortium is discussed as an opportunity to lay the groundwork for a new UV commercial industry, as well as the supply chain to support this industry. Several successful examples of consortia are applicable to the UV industry, and a hypothetical model is presented as an example that could help accelerate the establishment of uniform performance standards, especially as new technologies are being developed with wavelengths other than 254 nm. (Article to be published.)

Quantitative elucidation of biological inactivation by UV radiation is indispensable for understanding the functional application of UV sources in mobile or stationary disinfection devices and the molecular changes they cause. Characterization of the structural, energetic, and dynamic aspects of UV interactions with physical and biological media is essential for developing guidance, standards, and regulations for UV disinfection technologies. The optical interactions are not confined to small molecules or macromolecules, but instead apply to all surfaces in a whole-room environment with many surfaces and dimensions. In this special section, expert researchers in the field detail many of the perspectives and methods that are now being used to study and implement the use of UV technologies for disinfection, especially in light of the severe acute respiratory syndrome coronavirus 2 (SARS-CoV-2), the virus that causes coronavirus disease 2019 (COVID-19). These efforts include methods and approaches for experimental and theoretical analyses of the properties of physical and biological materials interacting with UV radiation. Papers include introductions to their respective topics, descriptions of the necessary materials and data for experimental studies, step-by-step laboratory protocols or suggestions, and key tips for understanding and measuring dose, efficacy, and materials effects.

These papers are the result of extensive multistakeholder engagement with the National Institute of Standard and Technology (NIST) and the International Ultraviolet Association (IUVA) beginning in 2017 (Table 2). These engagements led to a NIST–IUVA workshop in January 2020 to examine standards and metrology needs for UV disinfection technologies and applications to healthcare-associated infections (HAIs) and beyond. This introductory paper summarizes these engagements, the workshop, and subsequent work. Given the emergency of the COVID-19 pandemic in early 2020, the use of UV technologies for disinfection has grown in healthcare settings, but also in other sectors such as the buildings industry and the transportation sector. The needs for UV measurements, standards, and data have been evolving since early 2020 and now require greater attention to keep pace with novel UV disinfection applications and markets. By considering the emergence of new efforts to explore and use UV for disinfection, all the papers in this section provide information to help overcome challenges and support implementation strategies to meet the growing needs and demands for UV disinfection applications. Accordingly, this section balances global perspectives and interests in stimulating scientific discovery, protecting human health and safety, and promoting the growth of private sector activities. NIST recognizes its role as a leader in the development of internationally accepted measurements and data practices addressing emerging technologies, particularly with respect to UV radiation chemistry and physics, and is actively pursuing collaboration with stakeholders in industry, academia, health service organizations, and other agencies.

**Table 2:** NIST–IUVA engagements for developing measurements and standards for UV disinfection technologies in healthcare.

Activity and Date	Size of the Event	Stakeholders	Key Messages
NIST dialogue with industry, September 2017; see Sec. 4.1	Small (<25 people)	Industry, academia, government	A lack of standardized industry metrics for evaluating biological efficacy of UV-C devices is inhibiting the widespread adoption of UV-C disinfection technologies, and approaches to solve this barrier are needed.
Engagement with stakeholders at the IUVA 2018 Americas Conference, March 2018; see Sec. 4.2	Medium (50–100)	Industry, academia, healthcare services, and government	A multistakeholder working group is needed to coordinate development of guidance and best practices for manufacturers.
Formation of a stakeholder working group, March 2018; see Sec. 4.3	Large (>100)	Industry, academia, healthcare services, and government	An IUVA healthcare working group will be essential for coordinating the development of global guidance standards for evaluating UV-C device performance and efficacy.
Stakeholder workshop at Yale School of Medicine and Yale New Haven Health, September 2018; see Sec. 4.4	Large (>100)	Industry, academia, healthcare services, and government	Implementing standards and science-based metrology will support growing UV-C device deployment opportunities for improved public health and ensure product efficacy through requirements.
Engagement with stakeholders at the IUVA 2019 World Congress, February 2019; see Sec. 4.5	Large (>100)	Industry, academia, healthcare services, and government	Metrology solutions will require the best of government, industry, and academic thinking to identify key technology opportunities and challenges to routinely deploy and safely operate UV-C technologies in healthcare settings.
Stakeholder workshop by NIST–IUVA on UV technologies for healthcare and metrology needs, January 2020; see Sec. 5	Very large (150)	Industry, academia, healthcare services, and government	A set of recommended research directions was developed to further UV-C device deployment for greater public health, innovation, and global security.
Recapitulation and updates at the 2020 IUVA Americas Conference, March 2020; see Sec. 6.1	Large (>100)	Industry, academia, government	Further action toward the research directions from the NIST–IUVA workshop will help to address approaches to measure (a) UV disinfection of whole-room environments; (b) dose and efficacy of new UV sources such as light-emitting diodes (LEDs); and (c) UV inactivation of coronaviruses.
Technical perspectives from industry via a public webinar, September 2020; see Sec. 6.2	Very large (500+) virtual attendees	Industry, academia, healthcare services, and government	Due to the COVID-19 pandemic, technology development is outpacing guidance, standards, and regulatory developments, and many hospitals are adopting UV solutions without proper oversight in the absence of well-established standards. Data-driven decentralization of practices, where infection prevention is approached from multiple angles while leveraging statistics based on measurements and machine-learning, is needed.
Engagement with stakeholders at the International Conference on UV Disinfection for Air and Surfaces, December 2020; see Sec. 6.3	Very large (500+) virtual attendees	Industry, academia, healthcare services, and government	The collective development of guidance and best practices for measuring UV-C device efficacy is clearly necessary to build the foundation for industry standards development, especially in emerging technologies such as UV-LEDs and far UV-C (200 nm to 230 nm).
Industry perspectives via a public webinar on enhancing the new normalcy with UV disinfection, April 2021; see Sec. 6.4	Very large (500+) virtual attendees	Industry, academia, healthcare services, and government	Restarting normal activities following the COVID-19 pandemic can leverage UV-C disinfection strategies and implementation in the public, commercial, and residential buildings industry and the transportation sector. Peer-reviewed research publications are essential to help support development of guidance, standards, and regulations for UV disinfection technologies.

## 2 Healthcare-Associated Infections

The modern healthcare setting is a busy and complex environment with a dynamic exchange of patients and personnel no matter the locale. The setting, coupled with many types of invasive instruments, devices, and procedures to treat patients and help them recover, is continuously under threat from contamination by viral, bacterial, and fungal pathogens that multiply and spread via contact and airborne transmission. Infections can be associated with the devices used in medical procedures, such as catheters or ventilators. The resulting, iatrogenic, HAIs, also known as hospital-acquired infections or nosocomial infections, are those that are not initially present or incubating when healthcare is initiated but that subsequently develop in relation to healthcare exposure. These include central line-associated bloodstream infections, catheter-associated urinary tract infections, ventilator-associated or hospital-acquired pneumonia, and surgical site infections [1]. While *Clostridioides difficile*, methicillin-resistant *Staphylococcus aureus* (MRSA), and vancomycin-resistant *Enterococcus* species (VRE) are well-known bacteria that cause HAIs [2], infection from pathogens with additional antimicrobial resistance is an ever-increasing threat compounded by slowness in antibiotic development (Table 3), as predicted 20 years ago at the start of the new millennium [3]. In addition, viral and fungal resistance continue to emerge while parasites, zoonotic pathogens, and major shifts in traditional respiratory pathogens continue to create challenges. The public outbreak of SARS-CoV-2 and the efficiency of transmission for any respiratory virus have important implications for containment and mitigation strategies [4]. COVID-19 is not only a new biological concern [5], it has substantially impacted traditional HAI surveillance and prevention efforts [6]. The U.S. Centers for Disease Control and Prevention (CDC) [7, 8] and the World Health Organization (WHO) [9, 10] continuously monitor and work to prevent HAIs because they are a critical threat to patient safety and global health security.

**Table 3:** Examples of multidrug-resistant organisms (MDR) [11].

Abbreviated Name	Description
MRSA	Methicillin-resistant *Staphylococcus aureus*
VRE	Vancomycin-resistant *Enterococcus* species
CPE	Carbapenemase-producing Enterobacteriaceae
ESBLs	Gram-negative bacteria that produce extended spectrum beta-lactamases
MDR *E. coli*	Multidrug-resistant *Escherichia coli*
*KPC*	*Klebsiella pneumoniae* carbapenemase
*AB*	*Acinetobacter baumannii*
*S. maltophilia*	*Stenotrophomonas maltophilia*

**Fig. 1:**
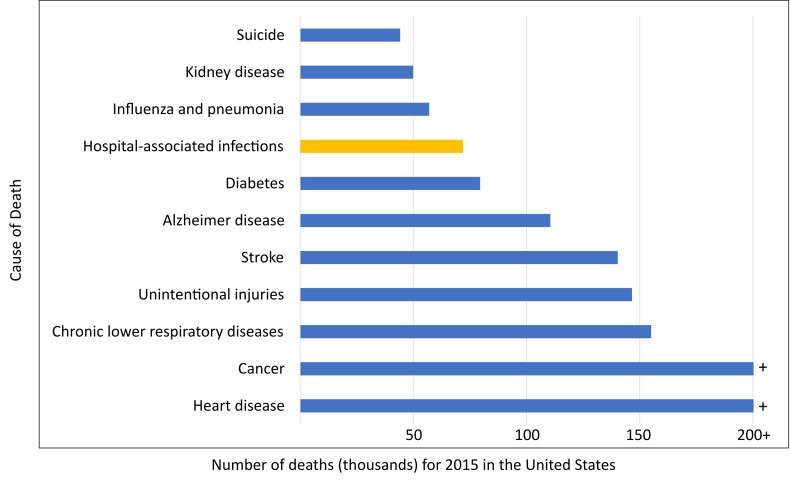
Number of deaths for the leading causes of death for the total population of the United States in 2015 compared to hospital-associated infections (bar in yellow), with heart disease and cancer being the top two causes of death, with 633,842 and 595,930 deaths, respectively (capped at 200,000 on the scale of the graph for illustration purposes). Data from Refs. [13–15]).

HAIs are now well understood to be largely preventable, and the broader medical profession has recognized that reducing pathogens in the healthcare environment has a significant impact on the risk of developing a HAI. In current HAI data reports, the CDC reported approximately one in 31 patients nationwide has at least one infection in association with his or her hospital care, underscoring the need for improvements in patient care practices in U.S. healthcare facilities [1]. In 2002, the estimated number of HAIs in U.S. hospitals, including federal facilities, was approximately 1.7 million, with 98,987 deaths [12]. In 2015, the most recently available published numeric data, the estimated number of HAIs in U.S. hospitals dropped to 687,000, but the number of deaths remained high at 72,000 [13, 14]. This placed HAIs as the eighth leading cause of death in the United States, just after diabetes (79,535 deaths [15]) at that time (Fig. 1). The cost estimates for hospital-onset HAIs from varying cost perspectives, including the economic burden to the U.S. healthcare system and excess payments made by insurers such as Medicare, are enormous but also widely vary [16]. While previous studies by healthcare providers have estimated an economic burden of approximately $10 billion annually for HAIs, a more contemporary examination of the impact of societal costs included the economic value of mortality risk reductions, thereby giving an estimate for the total economic burden to society in excess of $200 billion annually [16].

**Fig. 2:**
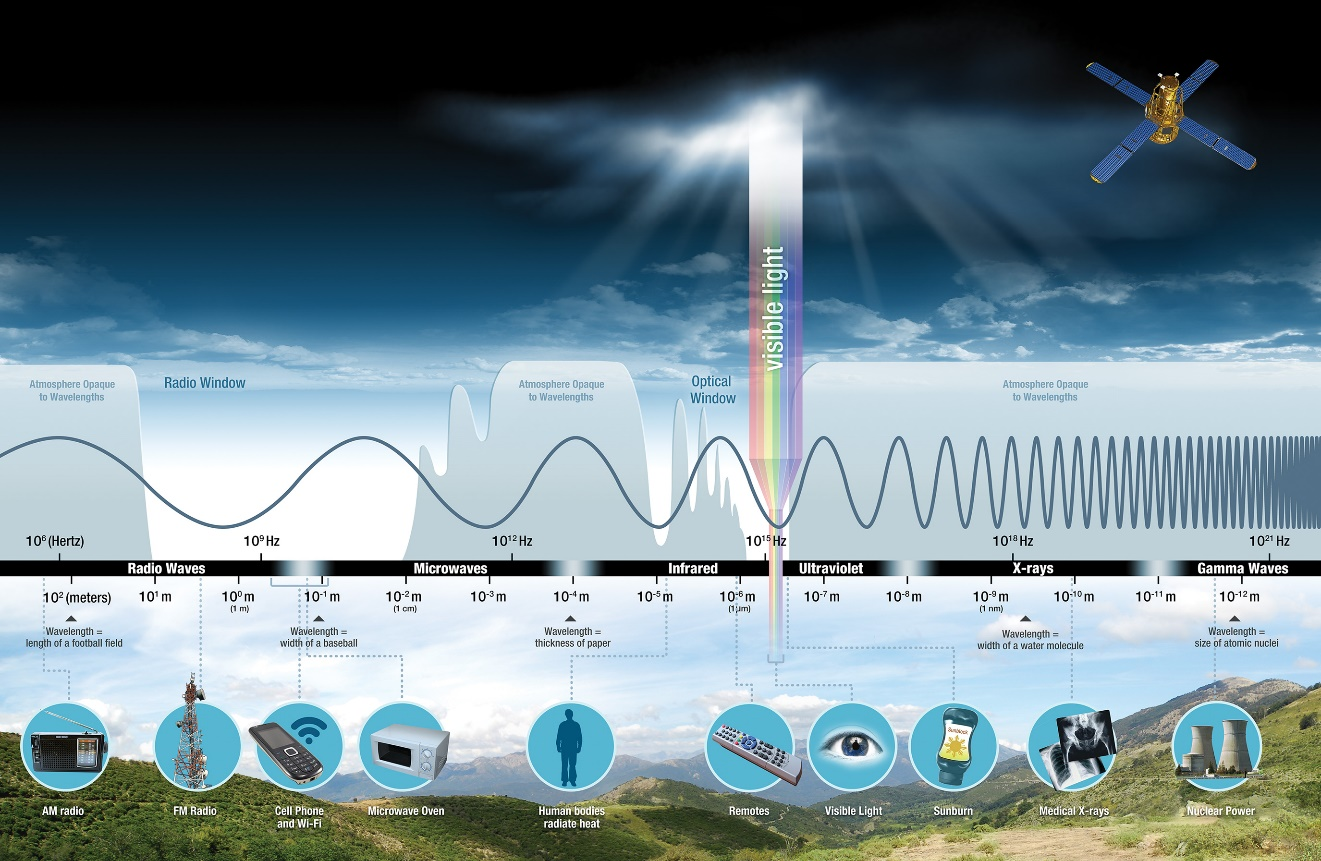
The sun’s electromagnetic energy travels in waves and spans a broad spectrum from very long radio waves (left) to very short gamma rays (right), with most waves being filtered by Earth’s atmosphere, except in the radio and optical windows, where the atmosphere is not opaque to those wavelengths. The human eye can detect only a small portion of this spectrum, called visible light. UV radiation is a very small portion of the sun’s electromagnetic spectrum, and the portion that disinfects does not transmit through the small atmospheric window shown for the UV region [27]. Figure credit: Science Mission Directorate, National Aeronautics and Space Administration (NASA).

Over the last decade, UV radiation^2^2 UV radiation is the term preferred at NIST to describe the energy emitted in the UV portion of the electromagnetic spectrum (100 nm to 400 nm). UV radiation should not be considered synonymous with UV light. The use of the term “UV light” leads to confusion due to the name of the visible region of the electromagnetic spectrum being “visible light” (see Fig. 2). has been shown to be an effective technology for infection prevention and control in the healthcare environment [17–24]. This stems from the well-understood use of UV radiation since the early 1900s to successfully disinfect water and the advent of artificial sources to generate the radiation at the proper wavelength to achieve UV disinfection [25]. This technology represents a global market valued at $4.8 billion for 2021 [26]. We discuss water disinfection in more detail in Sec. 3 and its proven success for the UV industry.

Modern UV disinfection technologies are clearly effective at reducing microbial contamination in healthcare environments. As an example, in an applied case study more than a decade ago making use of UV radiation to disinfect hospital room surfaces, the frequency of positive *C. difficile* environmental cultures was reduced by 80%, and MRSA and VRE environmental cultures were reduced by 93% [17]. However, progressing from case studies to widespread adoption has been difficult for the industry. There are key measurement and modeling barriers to deploying and safely operating UV technology in healthcare as compared to the wastewater industry.

It is difficult to compare wavelength and energy intensity among devices. An understanding of the operational needs among the wide variety of settings in a hospital environment is required. For example, device parameters such as distance from the target, duration of target exposure, and orientation of the surface being disinfected relative to the radiation source need to be specific to the radiation (energy) output of a device. In addition, the intrinsic susceptibility of the various microorganisms needs to be considered, among many other factors that are discussed below.

It is well understood by the UV industry and the healthcare community that commonly defined measurements can lead to consensus-based measurement standards for the widespread adoption of UV technology to healthcare applications. Standards development can be achieved through greater operational and fundamental understanding of the interactions of UV energy with biological and physical matter and the corresponding UV radiation physics and chemistry, which are active areas of research at NIST. The UV industry has been engaged with NIST for several years to identify key themes and technical challenges for UV technology commerce in healthcare, with the goal of informing NIST’s efforts in UV metrology for application to identified challenges.

In recognition of the rapid growth in UV commercial product development and use for preventing HAIs from occurring and possibly even treating some infections in patients, NIST, IUVA, IUVA member companies and affiliates, and academic collaborators have engaged to examine how the community can work toward developing standards and new metrology for UV testing, evaluation, and deployment in healthcare settings to best serve the needs of patients and public health. These interactions began in 2017 and resulted in an international NIST–IUVA UV metrology workshop in January 2020, and they continue into 2021. This paper summarizes these engagements with an emphasis on current activities in response to the needs identified at the January 2020 workshop.

## 3 Background on UV Radiation

UV radiation for applications in healthcare and wastewater should not be confused with the naturally occurring UV electromagnetic radiation that comprises approximately 10% of sunlight at ground levels. Electromagnetic radiation from the sun spans a broad spectrum from very long radio waves to very short gamma rays, which are reflected or absorbed by gases in Earth’s atmosphere. Among the most important gases are water vapor, carbon dioxide, and ozone. Some radiation wavelengths, such as long radio waves, a small portion of infrared red, visible light, and minute portions of UV radiation, are transmitted through the atmosphere to ground levels (Fig. 2). The regions of the spectrum with wavelengths that can pass through the atmosphere are referred to as “atmospheric windows” [27]. When captured and concentrated with heat, naturally occurring UV radiation at ground level can be used to inactivate microorganisms in water, a technique used throughout the developing world to disinfect water in plastic or glass bottles on a daily basis [28].

**Fig. 3:**
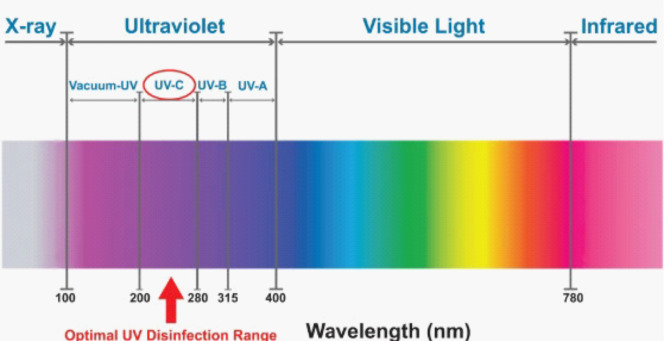
The electromagnetic spectrum from the sun, which includes various types of UV and other electromagnetic radiations and highlights the region for the optimal UV disinfection range [29]. Figure credit: Division of Technical Resources, Office of Research Facilities, U.S. National Institutes of Health.

**Fig. 4:**
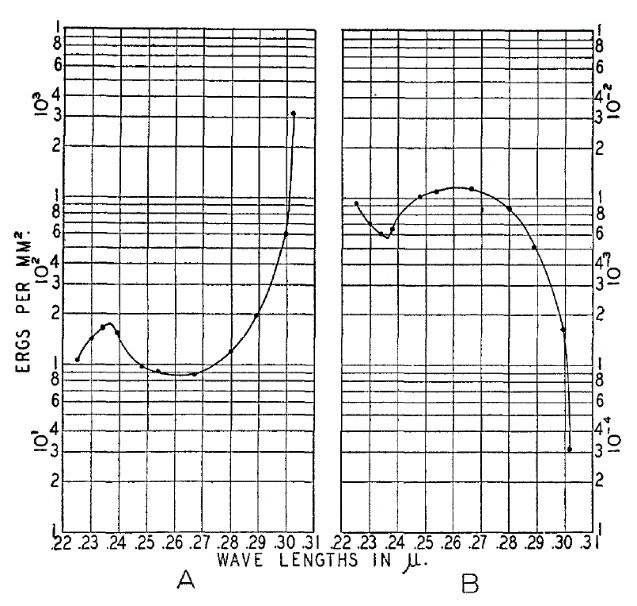
Historical measurements of the bactericidal action of UV radiation published by Gates in 1930 [39] using “methods of isolating and measuring monochromatic radiations, of preparing and exposing the bacteria, and of estimating the effects of exposure” [40]. (A) Curve of incident energies involved in the destruction of 50% of *S. aureus*. (B) Curve of the reciprocals of A. The curve on the left is described as a demonstration that “less incident energy is required between 260 nm and 270 nm than in any other region of the bactericidal zone examined and point toward a second minimum below 230 nm. The presence of a sharp peak in the energy requirement near 240 nm appears to be equally significant” [39]. Used with permission.

**Fig. 5:**
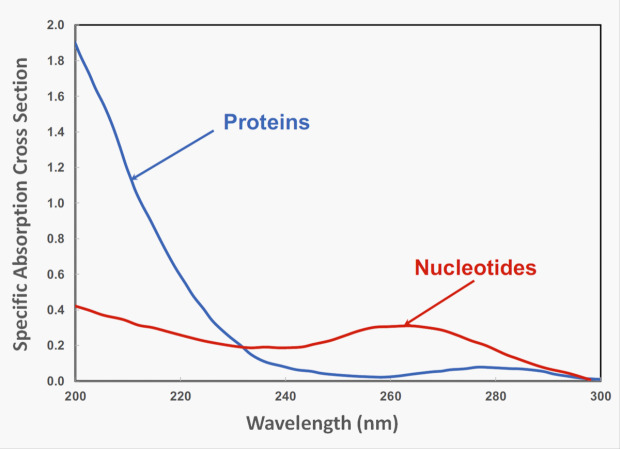
Specific absorption cross section versus wavelength for proteins and nucleotides within the cell of a microorganism. Here, the dry weight percentages are protein (70%), nucleotides (20%), and other components (10%) (*e.g.*, lipids). This figure was constructed per Bolton and Cotton [25] using average spectra for proteins and nucleotides along with the respective mass ratios.^3^3 See Harm W (1980) *Biological Effects of Ultraviolet Radiation* (Cambridge University Press, Cambridge, UK). Used with permission.^4^4 Bolton J, Poster D, American Water Works Association (2021) Personal communication.

**Fig. 6:**
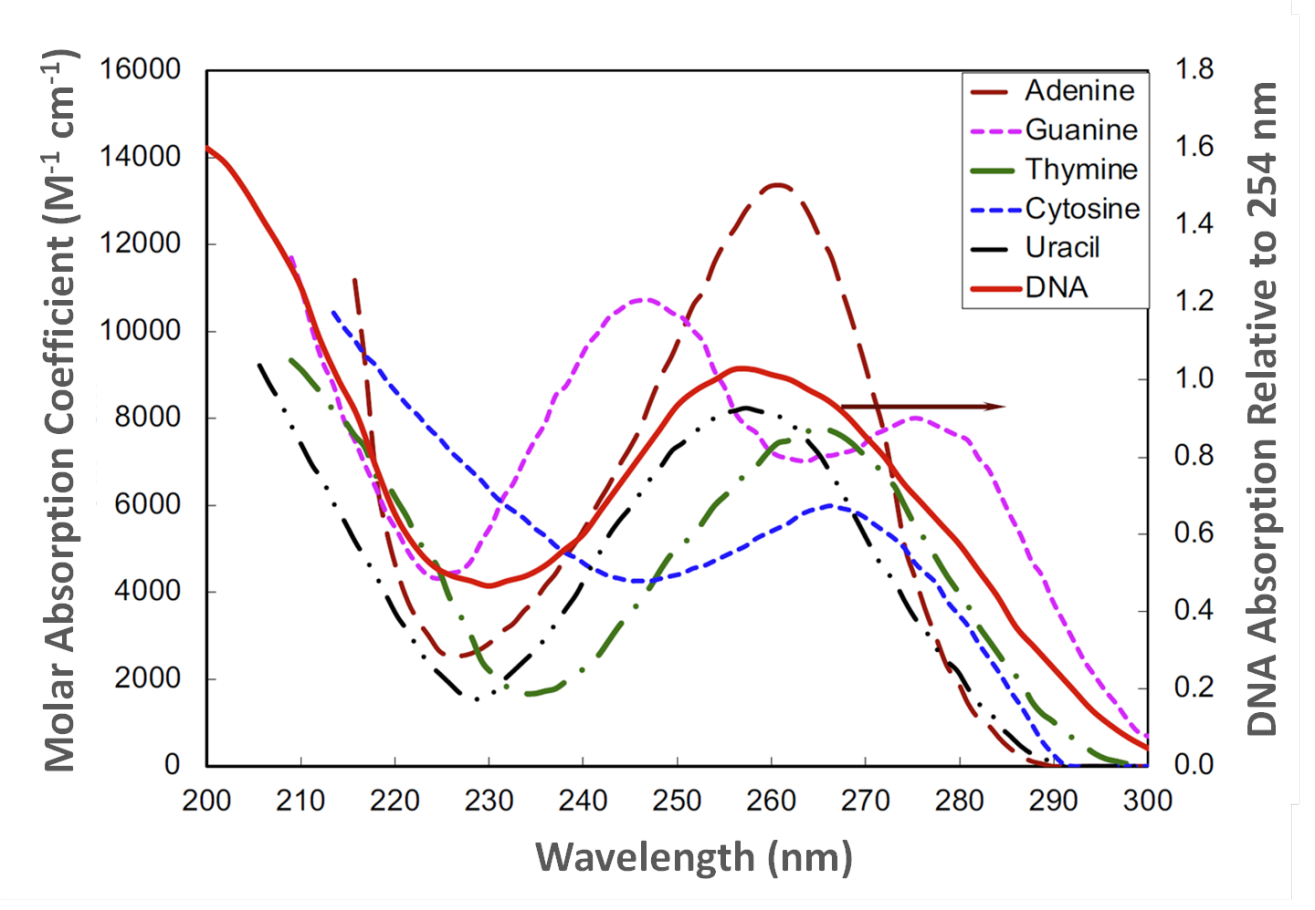
Absorption spectra of nucleotides and of DNA (from Bolton and Cotton [25], adapted from Harm, 1980^3^). Used with permission.^4^

There are four regions of UV radiation within the electromagnetic radiation spectrum. The four regions are classified based on the wavelength of the emitted energy wave measured in nanometers (nm), with the shortest wavelength in the vacuum-UV (100 nm to 200 nm), followed by UV-C with wavelengths between 200 nm and 280 nm, followed by medium-length UV-B with wavelengths between 280 nm and 315 nm, and ending with the longest wavelength UV-A with wavelengths between 315 nm and 400 nm [25, 29] (see Table 4; Fig. 3). The UV-C region is the effective region for inactivating microorganisms, such as bacteria and viruses, and it is often referred to as the “germicidal” region, since it can be absorbed by components of the microbe that makes them inactive, unable to multiply, and no longer able to cause disease [25], as we discuss briefly later in this section (see Figs. 4–6). X-rays (<100 nm in Fig. 3) are not germicidal. Energy with wavelengths shorter than 100 nm is filtered by the atmosphere (Fig. 2). X-rays in medicine and other applications must be generated by a source and are referred to as ionizing radiation, because, due to their short wavelengths and very high energy, the energy can split molecules and produce free electrons and reactive radicals. These cause biological disruptions at the molecular level, similar to the effects of radioactive source exposure [25]. This behavior is not comparable to microorganism inactivation resulting from the absorption of radiation; it is instead a reactive process. With that said, some UV radiation sources, such as Xe_2_ excimer lamps (see Table 5), emit shorter wavelengths. These have enough energy in the 172 nm range for the successful disinfection of air and water through chemical reactive mechanisms catalyzed by free electrons and reactive radical species generated by UV photolysis of water. This is not comparable to microorganism inactivation due to adsorption of radiation by components of the microbe [25].

**Table 4:** Description of the UV regions covering the wavelength range 100 nm to 400 nm in the electromagnetic spectrum [25, 29, 30].

Region	Description
Vacuum UV 100 nm to 200 nm	Shortest-wavelength UV-C: It is completely filtered by Earth’s atmosphere via absorption by oxygen in the atmosphere and does not reach Earth’s surface. Owing to atmospheric absorption, electromagnetic waves in this region can propagate only under vacuum and have been used since the 1960s in high-technology applications in the space sciences, high-energy physics, and electronics [31].
UV-C 200 nm to 280 nm	Shorter-wavelength UV-C: It is completely filtered by the atmosphere and does not reach Earth’s surface. Energy emitted in this range from UV radiation sources (Table 5) can be used for bacterial and viral inactivation due to absorption of the energy by protein and nucleotides comprising deoxyribonucleic acid (DNA) and ribonucleic acid (RNA) (see Figs. 4−6). This region is often referred to as the germicidal region [25].
UV-B 280 nm to 315 nm	Medium-wavelength UV-B: It is mostly filtered by the atmosphere via absorption by ozone, with about 0.1% reaching Earth’s surface at noon time at the equator in the summer [32]. It cannot penetrate beyond the superficial skin layers, but it influences the cutaneous production of vitamin D.^a^ However, with lengthy exposure, it can lead to delayed tanning and burning and promote skin aging and skin cancer. The portion of the spectrum between 280 nm and 300 nm can pass through a microorganism and be absorbed by various components in the cell, *i.e.*, mainly proteins and nucleotides (see Figs. 5 and 6) [25].
UV-A 315 nm to 400 nm	Longer-wavelength UV-A: It accounts for approximately 95% of the UV radiation reaching Earth’s surface because it is not absorbed by atmospheric ozone. It can penetrate the deeper layers of the skin and causes the immediate tanning effect. UV-A does inactivate bacteria, albeit much more slowly than UV-C [33].

^a^ Vitamin D is essential to human health by helping the body absorb calcium and phosphorus from food and assists in bone development. The WHO recommends 5 to 15 min of sun exposure two to three times per week to achieve these benefits [34]. The factors affecting vitamin D synthesis from UV-B radiation are discussed in Refs. [35, 36].

Energy in the visible region (400 nm to 780 nm; Fig. 3) and near-infrared region (780 nm to 1000 nm; Fig. 3) transmits through small atmospheric windows (Fig. 2) but is not considered germicidal, with the exception of 405 nm light [37]. Visible light provides energy input for growth, *i.e.*, photosynthesis in green plants and algae [25]. Energy in the near-infrared region has been shown to stimulate photosynthetic bacteria to store solar energy at wavelengths up to 980 nm [25], and energy beyond that wavelength up to 3 μm is demonstrating promise as input for biological therapies in neurostimulation, regeneration, and photodermatology [38], but it is not germicidal.

As the germicidal region of the sun’s UV spectrum is filtered out by the atmosphere, germicidal UV-C radiation must be generated and emitted from an artificial source. For more than a century, the germicidal influence of light on microorganisms has captured the interest of technologists aiming to generate the light artificially and apply engineering solutions to abate pathogen spread and disease. Two key advances were the development and commercialization of the low-pressure mercury (Hg) lamp in the early 1900s, followed by the discovery, in 1930, of the bactericidal action spectrum with a peak effectiveness at 260 nm to 265 nm (at which DNA absorbs the most energy), very near the 254 nm output of low-pressure Hg germicidal lamps [25] (Fig. 4).

As described by Bolton and Cotton [25], modern research has led to the understanding that UV-C radiation is germicidal due to the absorption of UV photons by microorganisms. The outer cell membranes can be disrupted by this absorption process, which leads to the demise of the cell due to leakage of the protoplasm, but only at very high doses that would be beyond typical UV-C radiation conditions for germicidal applications. Absorption of UV photons from UV-C radiation for germicidal application is by proteins and the nucleotides comprising DNA, or RNA in some viruses, and this leads to DNA or RNA structural changes, subsequently diminishing the ability of the microorganisms to replicate and as such become inactivated. Contemporary measurements of the adsorption of UV radiation by proteins and nucleotides are shown in Fig. 5.

Below 230 nm, where the energies of the radiation are higher, the proteins absorb most of the energy. These energy ranges are outside the typical UV-C radiation conditions for germicidal applications. Above 230 nm, adsorption is dominated by nucleotides. Between 200 nm to 300 nm, all the nucleotides in DNA or RNA (adenine, guanine, thymine, and cytosine in DNA; adenine, guanine, cytosine, and uracil in RNA) absorb UV radiation (see Fig. 6). Thymine is the fundamental nucleotide that leads to inactivation of a microorganism, because when thymine absorbs a photon, it forms pyrimidine dimers between adjacent thymine bases (these are also called thymidine dimers). For viruses, which do not contain thymine, dimerization of adjacent uracil bases also causes inactivation. Dimers disrupt the structure of a microorganism’s DNA, causing it to be unable to reproduce. When it can no longer reproduce, the microorganism can no longer cause infection [25]. Understanding the inactivation as a function of wavelength (energy) is essential. See the publication by Masjoudi *et al*. [41] for the current knowledge on the sensitivity of microorganisms to UV radiation.

Currently, four different types of lamps that produce and emit UV-C radiation are on the market (Table 5) with peak wavelengths within the germicidal segment of the electromagnetic spectrum [42]. Investigations of the use of UV-C radiation for infection prevention and control should consider the energy interactions among several different parameters, notably, time of exposure, lamp position in relation to the target surfaces or whole-room environment, barriers between the light source and target surfaces, intensity of emitted light, and extent and flow of air movement [43]. Boyce and Donskey [24] recently reviewed the physical aspects of UV-C radiation that affect the doses delivered to surfaces, the UV-C doses needed to yield reductions in several important HAI pathogens, the doses of UV-C that can be achieved in various locations in patient rooms using mobile UV-C devices, and the methods for measuring UV doses delivered to surfaces. The review by Spicer [44] provides an overview of the methods and mechanisms of photonic disinfection.

**Table 5:** Lamps that produce UV-C radiation and examples of healthcare applications.

**Low-pressure mercury lamp:** This is historically the most common type of lamp used to produce UV-C radiation, which has >90% emission at 254 nm. Other wavelengths are also produced by this type of lamp. There are other lamps that emit a broad range of UV wavelengths, including visible and infrared radiation. Rutala *et al*. [45] demonstrated the deployment of mobile devices equipped with low-pressure mercury lamps to produce continuous UV-C with a peak wavelength of 254 nm.
**Excimer lamp or far UV-C lamp:** These lamps provide high intensity in the UV or vacuum-UV regions (126 nm to 354 nm) of the electromagnetic spectrum generated by decaying excimer complexes formed in various nonequilibrium discharges; with different fill gases, narrow-band radiation at various UV and vacuum**-**UV wavelengths can be obtained [46]. The Xe_2_ excimer lamp has been used widely in the photochemical purification of air and water using advanced oxidation processes and taking advantage of the peak emission at 172 nm to trigger the photochemistry [46]. For microorganism inactivation on surfaces, the KrCl lamp is gaining attention due to its peak emission at 222 nm [47]. These lamps hold promise to be less dangerous for skin and eye exposure, although spectral characterization is important to detect possible minor emissions at longer wavelengths (above 230 nm may require optical filtering) [47]. Buonanno *et al.* [48] reported the efficient and safe use of 222 nm KrCl excimer lamps to inactive airborne human coronaviruses.
Pulsed xenon lamps: These lamps, which emit a short pulse of broad-spectrum light (including infrared radiation, visible light, and UV radiation), have been filtered to emit mainly UV-C radiation and are sometimes employed in hospital settings to treat environmental surfaces in operating rooms or other spaces. Casini *et al.* [49] reported the effective use of a pulsed xenon-based UV light no-touch disinfection system for the reduction of hygiene failures and control of environmental contamination by high-concern microorganisms such as *C. difficile* spores and KPC.
**Light-emitting diodes (LEDs):** These lamps produce UV radiation in a very narrow wavelength band of radiation. Currently available UV-LEDs have peak wavelengths at 265 nm, 273 nm, and 280 nm, among others (LEDs can be tailored to produce specific wavelengths). One advantage of LEDs over low-pressure mercury lamps is that they contain no mercury; however, the small surface area and higher directionality of LEDs may be disadvantages for UV-LED use in healthcare applications. In other applications areas, Rattanakul and Oguma [50] and Kim *et al*. [51] have demonstrated the effectiveness of UV-LEDs to inactivate pathogenic species in water and food, respectively. Also see the review by Spicer [44].

## 4 NIST and Community Engagement for the Development of UV Efficacy Standards

### 4.1 Dialogue with Industry on NIST Capabilities at the Gaithersburg, Maryland, Campus

On September 29, 2017, NIST hosted representatives of IUVA to discuss these topics and provide tours of the NIST UV laboratories and facilities. The group received an overview of NIST from the NIST Public Affairs Office and then met with leadership and researchers from the NIST Physical Measurement Laboratory’s Sensor Science Division and Radiation Physics Division, as well as the NIST Office of Advanced Manufacturing. NIST scientists and engineers learned about the current problems regarding HAIs, the state of UV technologies and applications to healthcare, stakeholder engagement, and challenges, gaps, and needs for the widespread realization of UV applications in healthcare.

During their visit, the group learned about the NIST Manufacturing Extension Partnership (MEP) [52], a public-private partnership with centers in all 50 states and Puerto Rico dedicated to serving small- and medium-sized manufacturers, and Manufacturing USA [53], a national network of manufacturing innovation institutes that advance the development of technologies to increase U.S. manufacturing competitiveness. The group also toured two NIST research facilities: the Spectral Irradiance and Radiance Responsivity Calibrations Using Uniform Sources facility, a reference laboratory for the calibration of detectors and radiometers for spectral irradiance responsivity and spectral radiance responsivity across the UV, visible, and much of the infrared spectrum; and the Synchrotron Ultraviolet Radiation Facility, a stable light source for radiometry and research.

Based on the presentations and discussions during this visit, participants concluded that the metrology needs of the manufacturers and users of UV-C devices for disinfection of whole-room environments are critical for the widespread adoption of UV-C technologies for pathogen abatement in healthcare settings. Metrology solutions would require the best of government, industry, and academic thinking to identify key technology opportunities and challenges to routinely deploy and safely operate UV-C technologies in healthcare settings. The collective development of guidance and best practices for measuring UV-C device efficacy is clearly necessary to build the foundation for industry standards development.

Activities subsequent to the visit included stakeholder education and outreach efforts via presentations by Cowan [54] and Martinello *et al.* [55] to the international biophotonics and biomedical optics communities for greater input on technology and metrology needs. These exchanges conveyed information about important needs and proposed pathways for developing consensus-based standards and methods for optical measurements and testing the efficacy of UV-C radiation antimicrobial devices and related technologies. Pathways from that point continued to encompass NIST, IUVA, and UV industry stakeholders, including clinical doctors and medical professionals from the local medical community and academic teaching hospitals and medical centers. It was clear that NIST photometry and biological metrology programs could provide essential input to the development of measurement approaches for assessing UV efficacy and that industry could benefit by having access to measurement capabilities, science, and data they might otherwise not have in isolation.

### 4.2 Engaging Stakeholders through IUVA’s 2018 Americas Conference

In February 2018, NIST and IUVA jointly organized a special conference session on UV technologies for combating HAIs through standards development at the IUVA 2018 Americas Conference in Redondo Beach, California. Several panels of subject-matter experts and attendees from industry, academia, healthcare services, and government participated in engaging and bidirectional information sharing between stakeholders on the current state of the science for UV technologies and applications in healthcare. A key message from the panels was that measurements of UV-C interactions with biological and physical matter are needed for healthcare settings to support the development of guidance and best practices for manufacturers and enable comparability among devices.

**Fig. 7:**
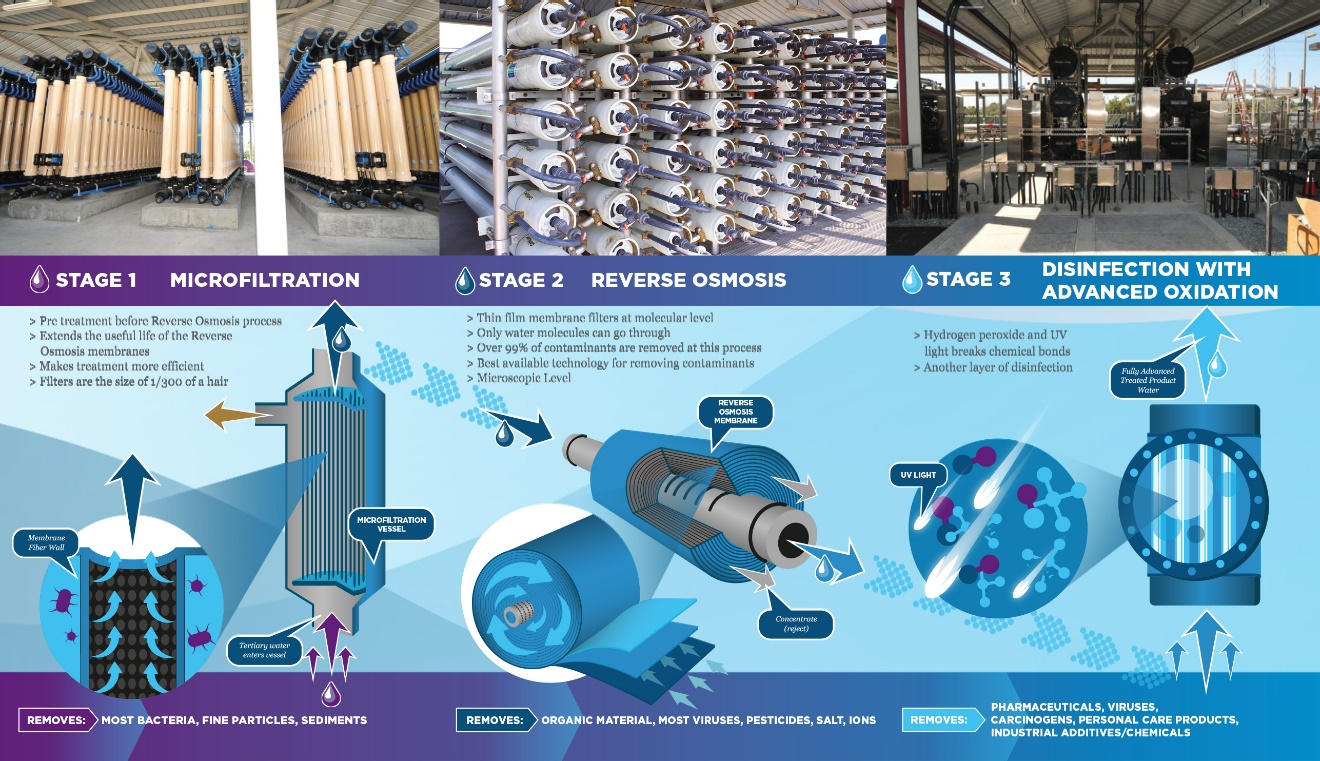
Advanced water treatment process at the Water Replenishment District of Southern California (WRD) Leo J. Vander Lans Water (LVL) Treatment Facility [57]. Stage 3 includes the use of UV radiation for the disinfection of microorganisms and other components that are broken down due to advanced oxidation processes catalyzed by photolysis reactions of water with UV radiation. Figure credit: WRD LVL.

An additional aspect of the Americas Conference was the engagement with the water treatment industry. UV-C devices have long been used in the drinking water and wastewater treatment industry for disinfection of waterborne microorganisms and are regulated by the U.S. Environmental Protection Agency (EPA). Participants from the Americas Conference had the opportunity to tour the Water Replenishment District of Southern California Leo J. Vander Lans Water Treatment Facility. The facility uses a combination of microfiltration, reverse osmosis, and UV disinfection with advanced oxidation for water treatment (Fig. 7), with a capacity to process 300,000 hectoliters (8 million gallons) per day. The facility recovers 92% of its wastewater intake to a near-distilled quality, making the facility a global leader in water efficiency. As was mentioned in Sec. 2, water disinfection using UV technologies dates to the early 1900s [25], and as described in Table 5, the Xe_2_ excimer lamp has been used widely in the photochemical purification of air and water using advanced oxidation processes, taking advantage of the peak emission at 172 nm to trigger the photochemistry.

IUVA leadership and NIST representatives also visited California Manufacturing Technology Consulting [56], the NIST MEP Center for the State of California, to learn about possible opportunities for UV applications in various other industries and manufacturing sectors in California, such as food and beverage, horticultural, materials science, aerospace engineering, and healthcare. These are important activities for understanding and furthering optical technologies and device capabilities for disinfection that require sophisticated and advanced measurements, standards, and data.

### 4.3 IUVA Healthcare Working Group

An output of the 2018 Americas Conference was the formation of a formal IUVA healthcare working group (WG) for the development of standards and initiatives for the deployment of UV in healthcare applications. The goal of the WG is to provide global guidance standards for evaluation of performance and efficacy of UV devices and to develop and guide a roadmap to address the needs and challenges for greater UV application in healthcare.

Standards development has been proven to have a pronounced effect on product development and the success of businesses in the marketplace. Implementing standards and integrating science-based metrology to ensure product efficacy through requirements either for trade or regulatory needs are critical for streamlining UV device manufacturing, increasing market penetration, and growing deployment opportunities for the improvement of public health. These concepts and the formation and successes of the WG since the 2018 event, including a standards roadmap for UV technologies in healthcare and collaborative models for engaging UV and public health stakeholders through novel partnerships, are discussed in the paper by Poster *et al.* [58], which discusses collaborative models that are ideal for engaging UV and public health stakeholders through novel partnerships to meet the needs of the standards roadmap.

NIST has been a member of the WG since its inception. To date, there are more than 100 members of the WG from industry, government, and academia. Several task forces have been established to develop guidance on the following topics: efficacy standards development (includes participation from the Illuminating Engineering Society [IES]); far UV-C (200 nm to 230 nm) [47]; UV-C LEDs; COVID-19; and consortium development [58]. The WG continuously discusses standards (see Sec. 7.1).

### 4.4 Workshop at Yale School of Medicine and Yale New Haven Health

In September 2018, the WG held a formal workshop at Yale University to collect information and discuss in detail the issues related to the proposed efficacy standards for UV disinfecting devices [59]. This event attracted more than 70 attendees from around the globe representing industry, academia, healthcare services, and leadership from NIST and the U.S. Departments of Health and Human Services, Energy, and Commerce. The workshop discussions focused on the impact of HAIs, the proven and hypothesized benefits of UV-C technology to prevent HAIs, and the need to improve the effective use of UV-C technologies for healthcare applications.

Highlighted concerns included the need to develop standardized methods to measure the dose of UV-C radiation required to inactivate pathogens relevant to the healthcare environment; the need to develop a more complete understanding for the ways in which pathogens that contaminate surfaces, air, and water in the healthcare environment result in HAIs; and the need to determine ways to effectively design and deploy UV-C to create a safer healthcare environment and prevent HAIs. Participants concluded that the next workshop should be held at NIST to explore in depth these research and metrology needs.

At the conclusion of the workshop, participants were given a tour of the Yale New Haven Hospital air ventilation systems equipped with UV technologies. Air systems equipped with UV technologies offer increased air quality to provide patients with a safe environment by reducing airborne transmission of microorganisms. The UV technology systems also contribute to meeting Leadership in Energy and Environmental Design (LEEDs) certifications used in the qualification of the modern “green” building rating system.^5^5 The U.S. Green Building Council (USGBC) created LEED to measure and define buildings that incentivize healthy work environments and efficient operations in the building industry [60]. The LEED rating system gives credit for air ventilation systems with UV technologies in the categories of energy and atmosphere, indoor environment quality, and innovation and design process. ASHRAE (formerly the American Society of Heating, Refrigerating and Air-Conditioning Engineers) works cooperatively with the USGBC to promote buildings that are healthful, environmentally responsible, comfortable and productive, profitable, and sustainable, and it provides educational materials on UV technologies for air and surface disinfection [61]. See Sec. 6.4 for information on a recent webinar by NIST and IUVA that discussed buildings and UV technologies. Yale New Haven Hospital is actively engaged in construction and renovation projects to improve energy efficiency, water conservation, air quality, and the work environment and is LEED certified for its efforts, which include UV technology applications for air quality.

### 4.5 Continued Community Engagement 2017–2020

In addition to the Yale University Workshop, NIST and IUVA have provided multiple presentations to the technical community over time, to the International Society for Optics and Photonics (SPIE) [54, 55, 62], the International Commission on Illumination (CIE) [63], the International Organization for Standardization (ISO) Technical Committee 142 [64], and the international delegates of the 2019 IUVA World Congress. The latter included plenary presentations by Dianne Poster *et al*. [65] and Richard Martinello [66] on the state of the field for UV-C radiation and HAIs and ongoing work within the community. A unique aspect of the 2019 IUVA World Congress was the engagement of the UV technology sector prevalent in southeast Asia, where UV applications are widely used for the drinking water, food, and beverage industries as well as in healthcare.

The NIST and IUVA 2017–2020 collaborative efforts and the ongoing events with the IUVA healthcare WG cumulated in an international metrology event at NIST in January 2020 (see Sec. 5). From January 2020 to the present, many informational briefings and collaborative efforts with organizations have taken place. For example, the IUVA has signed a memorandum of understanding with both the Illuminating Engineering Society (IES) and ASHRAE. Additionally, IUVA has been accepted into the U.S. Environmental Protection Agency’s Smart Sector Program and approved as a member of the U.S. Food and Drug Administration’s Network of Experts.

These engagements have been steady and tailored to gain input and share subject matter expertise on metrology, standards, data, and technology needs for UV-C healthcare applications, including a public World-Wide-Web–based seminar (webinar) in September 2020 and a partnership between NIST and IUVA for the 2020 First International Conference on UV Disinfection for Air and Surfaces conference, as well as a number of public webinars (see Sec. 6). All this engagement continues to further efforts to discuss, deliberate, and develop UV standards for the implementation of UV technologies to aid in the reduction and prevention of HAIs [54, 55, 62–69].

## 5 NIST–IUVA Workshop on UV Technologies for Healthcare and Metrology Needs

On January 14−15, 2020, NIST and the IUVA collaboratively hosted the International Workshop on UV Disinfection Technologies at the NIST campus in Gaithersburg, Maryland. The event drew 150 attendees from the public health community, with top medical, academic, and policy experts participating and international subject matter experts speaking, providing tabletop displays of technologies, and presenting posters.

The initial emphasis of the 2020 NIST–IUVA workshop was HAIs and the effectiveness of UV technologies on the targeted pathogens. However, the critical need was amplified with the outbreak of SARS-CoV-2, the virus that causes COVID-19. UV-C disinfection practices are quite efficacious, but adoption has been hindered due to a lack of education and awareness about UV-C. Worse still, fraudulently labeled, untested products have made their way into the marketplace, with little means of validating performance available to anyone. Failure to consistently embrace the technology is driven largely by uncertainty of the efficacy of the devices versus the cost to acquire the instruments and implement their technology [54]. Thus, additional research and motivation to overcome this disparity are critical for success, as well as involvement and education via conferences, workshops, and webinars about UV-C disinfection.

In this workshop, NIST and IUVA engaged with the community to explore ways to address these challenges and help to advance measures of device disinfection efficacy, reliability, and operation [70]. The workshop built on the efforts of the IUVA healthcare WG (see Sec. 4.3) dedicated to the development of efficacy guidelines and standards to help advance the adoption and safe use of UV-C technologies. This effort focused on examining measurement, standards, technology, and data research needs to promote innovation in the effective use and implementation of UV-C technology in healthcare settings for the reduction and prevention of HAIs. UV-C devices are known to reduce the incidence of HAIs (see Secs. 2 and 3), but the adoption of the technology by the healthcare industry has been sparse. Performance standards that would help healthcare managers make informed, credible investment decisions are lacking. That is, performance measurements and standards appropriate for UV-C radiation and biological efficacy are needed to accelerate the adoption of UV-C technology in the healthcare market. The rapid onset of the COVID-19 pandemic expanded the scope of the workshop to include the SARS-CoV-2 pathogen, although at the time of the event, the magnitude and impact of SARS-CoV-2 were unknown.

The latest developments in UV-C technology, engineering, and operations, and regulatory concerns related to disinfection were presented. Discussions on the need for standards in both the physical and biological performance areas for UV-C disinfection devices were central to the event. Papers in this special section of the *Journal of Research of the National Institute of Standards and Technology* highlight the perspectives of the workshop and detail the technical discussions.

During the event, measurements, standards, and data supporting UV-C disinfection were discussed at great length in the context of innovation and implementation to support public health and combatting infectious diseases. Speakers identified critical metrology and standards drivers, gaps, and impediments to UV-C technology adoption in healthcare, and potential benefits to individual patients and broader public health. Cowan [70] noted that these difficulties have been compared to industry’s success in the water treatment sector (see Sec. 4.2), resulting in questions such as, What lessons can we learn from UV in the water treatment market? Due to the solid surface requirements for disinfection in the healthcare environment, it is difficult to compare the two industries. One focuses on water (see Fig. 7), and the other focuses on variable solid surfaces with complex geometry. Also, water legislation and regulations have been well defined for many years, but healthcare applications have no legislation or regulations yet. These conversations have only just begun. UV water technologies focus on waterborne pathogens that are well-understood chemically, legislatively, and regulatorily. UV disinfection technologies in healthcare focus on entirely different types of pathogens that are less well defined in terms of their susceptibility to UV-C. Last, the targeted water pathogens are confined and processed in specific treatment streams that are well defined both from an engineering perspective and a chemical perspective. Target healthcare surface pathogens are dispersed, and mixed, throughout an entire facility and are not confined. Process control is specific to a locale rather than a facility, making metrics for performance measurements extremely difficult.

### 5.1 NIST’s Role

Remarks from Dr. Walter Copan, then the Under Secretary of Commerce for Standards and Technology and director of NIST, conveyed how NIST could provide a neutral, nonregulatory environment to build consensus for UV-C efficacy standards. NIST conducts measurements, develops standards, and performs technology research that helps build community-wide consensus on terminology, best practices, and risks for high-technology industries. Key stakeholders are brought together to work through the questions from many perspectives. Efforts are made to initiate difficult conversations, find consensus, and bring the public and private sectors together. This approach helps to ensure that the existing relevant standards and processes in the field are considered and then built upon. NIST does not dictate the outcome, but acts as an impartial, knowledgeable facilitator for the discussion and building of consensus. In the context of standards, consensus does not imply unanimity but is defined as all participants having the opportunity to express themselves and explain their positions in a collegial and collaborative environment. Consensus represents a decision all participants can accept, even if they have disagreements.

The mission of NIST is to work with industry, academia, and government to advance innovation and improve quality of life. It would be impossible to satisfy that mission without being a neutral, trusted, technically focused organization. A core value of NIST is to hold the highest standards of integrity and quality. NIST maintains the trust of stakeholders by attending to details and fostering a culture of openness and fairness. Much of the world’s industrial, engineering, and science base relies on NIST measurements, reference data, tools, and calibrations.

The development of important efficacy standards for treating infectious disease with UV-C technologies should be an open process where participants can meet with their colleagues, learn whom to consult, understand their peers’ activities and tools, and know how to make informed decisions. NIST recognizes that efforts to combat HAIs are for the public good. Work in this area on producing, managing, and generating research data, and developing new technologies will be essential to success. The work of this community is of national and global value.

Dr. Eric Lin, then director of the NIST Material Measurement Laboratory, also offered warm welcome to everyone and noted how the workshop was a unique and important opportunity for the community and NIST to connect and work together on the critical topic of HAIs and technological solutions through innovation.

### 5.2 Challenges for UV Technology Development and Deployment in Healthcare

**Fig. 8:**
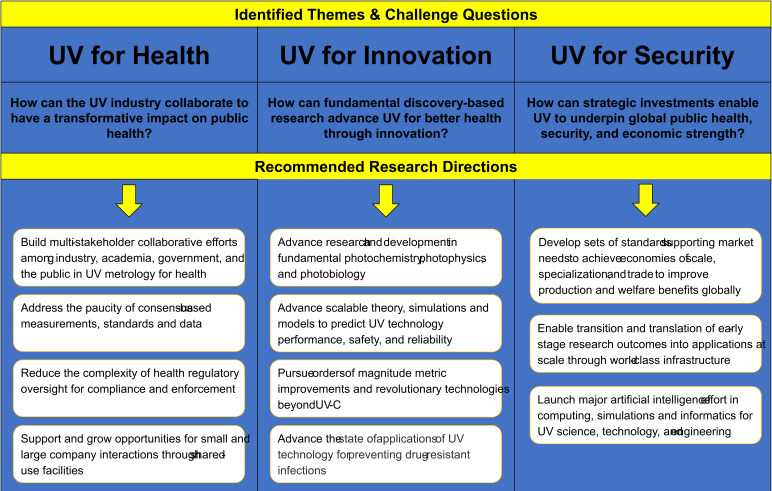
Recommended research directions from the NIST–IUVA workshop on UV technologies for healthcare, January 14−15, 2020, NIST, Gaithersburg, Maryland.

The workshop posed three major challenges with the distinct themes of health, innovation, and security (Fig. 8). These challenges were formulated to inspire advancements in the widespread use of UV technologies rapidly and effectively in healthcare applications for better public health and to combat the COVID-19 pandemic. Stated as challenge questions, these were:

• How can the UV industry collaborate to have a transformative impact on public health, including infectious disease control and prevention?
• How can fundamental discovery-based research, with support from federal science agencies, advance UV for better health through innovation?
• How can strategic investments in measurement equipment, standards development, advanced technology, and data analysis enable UV to underpin global public health, security, and economic strength?

### 5.3 Recommendations

Recommended research directions for the UV community were developed to answer the challenge questions posed in Fig. 8, with the goal of increasing adoption of UV technologies for pathogen abatement in healthcare environments. The findings below express the enthusiasm and excitement of the participants to apply UV in health, innovation, and global security.

#### 5.3.1 UV-C for Health

For UV-C to be successful in the healthcare environment, consumer confidence in UV metrology needs to be built through a multiple-stakeholder collaborative effort among industry, academia, government, and the public. Poster *et al*. [58] proposed an approach to that challenge: Consortium development helps to overcome the paucity of existing consensus-based measurements, standards, and data through mutual trust and collaborative funding models that expand precompetitive UV-C research and standards development. A legally recognized consortium can speak for all its member companies, providing a strong base for support. In addition, a consortium can seek ways to reduce the complexity of health regulatory oversight for compliance and enforcement. It can also help to correct misinformation and identify poor-quality products that have entered the market owing to a lack of stringent and uniform performance testing and standards, such as those that could be developed through consortium efforts. A consortium can support and increase opportunities for small and large company interactions and potentially develop shared-use buildings for further research. Donskey noted that healthcare facilities need practical methods to assess UV-C dosing [71]. Colorimetric indicators provide a rough estimate of UV-C doses and are promising because they are easy to use [72]. However, methods to assess UV-C efficacy require standardized testing protocols [71]. Specific factors that should be measured for validating UV-C devices for room disinfection were presented by Weber [73]: colonization of patients; infection in patients; confounders (hand hygiene compliance, cleaning compliance); cost; delays in admission to the room; and transmission pathways of microbes. Weber [73] also noted the important question, Are all UV room disinfection devices similar, or do we need validation of each device? A collaborative model to develop research programs with all stakeholders engaged is essential for meeting these complex needs.

#### 5.3.2 UV-C for Innovation

The participants felt it was important to update the existing body of research and development achievements in fundamental photochemistry, photophysics, and photobiology to bring the science to the forefront of applications. Few research studies are currently available on the efficacy of UV-C as a function of intensity, exposure time, modeling, surface and surface effects, and geometry. Further research into the application of UV-C technology to control and reduce antibiotic resistance is needed. It is well known that UV-C can inactivate pathogens (as described in Secs. 2 and 3), but additional research is needed in theory, simulations, and validated models to predict UV-C technology performance, safety, and reliability. This might require the pursuit of ambitious projects with orders of magnitude improvements and revolutionary technologies beyond UV-C.

Boyce [74] highlighted the need for additional studies of the ability of various UV devices to deliver doses that effectively reduce healthcare-associated pathogens using fixed and portable equipment located in patients’ rooms and other clinical areas and to assess other light modalities (e.g., 405 nm; continuous UV-A). Additional needs highlighted were: standardized methods for comparing device efficacy in order to assist facilities in choosing the most appropriate device; practical methods of monitoring the doses delivered, especially for devices without accompanying UV-C sensors; and additional prospective trials of the impact of UV radiation on HAIs using a variety of devices (along with a comparison of the impact of different devices and data on cost-effectiveness).

Self-disinfecting surfaces are of considerable interest for applications in healthcare settings. Roeder [75] discussed how photocatalysis offers innovative solutions using the common photocatalyst titanium dioxide (TiO_2_) as an example. Surfaces can be modified with an applied coating of TiO_2_, which is then activated by illumination to generate reactive oxygen species (ROS), which in turn can inactive microorganisms. Roeder [75] demonstrated the inactivation of *Bacillus subtilis* PS533 spores using a cast film of a TiO_2_-based photocatalyst with 365 nm light. This provides evidence that photocatalytic surfaces are a promising technique for inactivating pathogens. Photocatalyst-substrate design offers many innovative options for tailoring custom solutions to meet the illumination and biological needs for specific settings, such as a mobile surgical hospital laden with low illumination but high biological threat conditions.

#### 5.3.3 UV-C for Security

Development of performance standards is needed for the market to grow and thereby attain the economies of scale required for broad market adoption. This can be accomplished by enabling transition and translation of early-stage research outcomes into large-scale applications through world-class infrastructure, which could be facilitated through a consortium [58]. Swain [76] noted that new technology sources and the future landscape for them must consider factors that can lead to economies of scale and market growth such as technology maturity, cost effectiveness, reliability, energy efficiency (for energy security), and environmental compatibility (reuse or recycle capabilities). Research efforts that support artificial intelligence (AI) efforts in computing, simulations, and informatics for UV-C science, technology, and engineering can help to develop secure approaches to address these factors quickly and proactively.

In addition, innovation for security will require AI developments as technologies that foster UV radiation applications grow. For example, the effort to improve the effectiveness of UV-C room disinfection devices through new ancillary technologies, such as photocatalysis (discussed above, Ref. [75]) or reflective paints using nanotechnology, can benefit from AI approaches to better understand their design, chemistry, and effectiveness. Weber [77] described a prototype for a reflective paint/wall coating that has been evaluated in several studies conducted in unoccupied patient rooms using test surfaces contaminated with healthcare-associated pathogens. The researchers concluded: (1) UV-C reflective wall coatings significantly improve UV-C radiation intensity delivered to room surfaces, with greater improvement for indirect exposures; (2) UV-C reflective wall coatings significantly improve microbial inactivation with reduced exposure time to achieve similar inactivation levels; and (3) UV-C ceiling and/or floor coatings do not improve microorganism reduction when walls are coated. Computing, simulations, and informatics can support novel technology development such as this and will help in assessing the impact and costs of such applications. Examples include using the wall coating in hospital units (*e.g.*, an intensive care unit) and a cost analysis of the benefits of using reflective wall coatings with parameters such as cost of UV-C reflective coating versus standard paint, timing for reapplication versus that of standard paint, and number of rooms that needed to be covered [77] to enable the maximum health protection possible. In countries where energy requirements may be less and UV capabilities are available, both photocatalysis and reflective technologies can be very beneficial.

#### 5.3.4 Measures of Success

Measures of success could be enhanced multisector engagement in research and innovation, greater efforts in the application of discovery research to product development, a growing community of new researchers and practitioners, and better patient outcomes by the effective prevention of HAIs. The findings of the workshop below convey the enthusiasm and excitement of the private and public sectors to apply UV-C for health, innovation, and global security. The recommended research and development directions were identified as critical to achieving widespread use of UV-C for reduction and prevention of infectious disease and for enabling better control of antibiotic resistance worldwide. A “roadmapping” event is envisioned as the logical next step to continue the discussion on critical research and development challenges and solutions.

## 6 Outcomes of the Workshop

Several immediate outcomes from this successful collaborative event were:

• an elevated awareness of the advantages afforded by UV in disinfection technologies;
• a demonstration of the immense advantages of public-private endeavors among industry, academia, and government agencies in other areas;
• plans to recapitulate the workshop and cover updates at the 2020 IUVA Americas Conference, March 8–11, 2020, Orlando, Florida (see Sec. 6.1);
• plans to host a public webinar to provide a summary of the workshop and the latest technical perspectives (see Sec. 6.2); and
• the development of summary articles to help guide, inform, and fuel collaboration among industry and healthcare stakeholders to meet the challenges, needs, and opportunities in UV technologies for disinfection and the control of antibiotic resistance. Some of these are presented in this special section of the *Journal of Research of the National Institute of Standards and Technology* (see Sec. 1).

### 6.1 Recapitulation and Updates at the 2020 IUVA Americas Conference

A review and updates of the topics from the 2020 NIST workshop took place at the 2020 IUVA Americas Conference, March 8−11, 2020, in Orlando, Florida. Of special interest was a session titled “UV for Healthcare Applications,” in which presentations were provided by Cameron Miller, NIST (on behalf of Richard Martinello), titled “Defining Standards and Metrology Needs for Ultraviolet Disinfection Technologies and Healthcare-Associated Infections through Industry and Federal Collaboration: A Summary of the NIST/IUVA Workshop”; Arthur Kreitenberg, Dimer, LLC, titled “Can UV-C Disinfect Fabric Hospital Privacy Curtains?”; and Marvin Ruffin, Excelitas Technologies, titled “Preventing Hospital-Acquired Infections with UV-C Light-Emitting Diodes.” In addition, there were two panels with subject matter experts from industry, academia, and government agencies to discuss the latest development of efficacy standards for UV in healthcare and perspectives on UV technologies and the coronavirus. Key perspectives and recommendations regarding standards for UV-C whole-room disinfection were provided by Kreitenberg and Martinello [78].

### 6.2 Technical Perspectives from Industry via a Public Webinar, September 30, 2020

More than 500 attendees registered for the September 30, 2020, public webinar hosted by the IUVA in collaboration with NIST. Industry participants discussed their various perspectives on the COVID-19 pandemic and their respective organizations’ responses. The presenters also emphasized the difficulty of working within current regulatory structures and of operating without well-established standards. For instance, a presenter noted that while there was an increase in awareness of infection-prevention practices and, consequently, of UV-C technology for infection prevention, the sudden influx of UV-C devices into the marketplace revealed a lack of design controls in the broader industry. This indicates, of course, that UV-C technology designed for disinfection of surfaces in healthcare settings is still a relatively young industry.

Presenters relayed the clear increase of interest in UV-C technology specifically in the healthcare sector. Hospitals with shortages of personal protective equipment (PPE), especially N95^6^6 “N95” is a filter class designation of the U.S. National Institute of Occupational Safety and Health (NIOSH). It is applied to respirators that are at least 95% efficient at filtering NaCl aerosols with particle sizes of mean diameter 75 nm ± 20 nm (NIOSH Procedure No. TEB-APR-STP-0059, December 13, 2019). filtering facemasks, desired to use UV-C technology to disinfect and extend their supply. Multiple approaches to disinfection of PPE by UV-C technologies are being pursued by industry (and some were explained in the webinar discussions), and the CDC has issued guidance on using UV-C to disinfect N95 facemasks [79]. Still, discussants acknowledged that there is much more to learn in this area. Most notably, UV-C studies typically focus on nonporous surfaces, whereas disposable PPE is typically made of fibrous, porous material. In addition, material degradation was emphasized as an underappreciated aspect of UV-C disinfection protocols, and it should be studied more comprehensively. See papers by Geldert *et al*. [80] and Chandran *et al.* [81] for a discussion on PPE and N95 masks and the use of UV-C technology for disinfection of PPE and N95 masks and recommended best practices.

These industry participants universally agreed that healthcare workers are accepting and adopting UV-C technology more rapidly in the face of the COVID-19 pandemic, but more education for both healthcare workers and the public regarding UV-C’s effectiveness for preventing other HAIs is needed. The technology development is outpacing standards and regulations developments, and many hospitals are adopting solutions without proper oversight. The current regulatory environment makes a level of disinfection claim that is not applicable to the diverse sets of applications for which UV-C technology can be used. In addition, an agreed-upon panel of microorganisms relevant to healthcare surfaces is needed. Finally, participants made it clear that UV-C technology is an important part, but not the only part, of infection-prevention approaches needed to reduce HAIs more broadly. Data-driven decentralization of practices, where infection prevention is approached from multiple angles while leveraging statistics based on measurements and machine-learning, is needed to make significant progress. Even within UV-C technology solutions, a multipronged approach would be useful. Significant advances in understanding UV-C dose, such as development of mathematical models to estimate irradiance distributions from UV-C sources to surfaces and estimating doses [82], will be important. Also, see the significant reviews by Masjoudi *et al*. [41] and Blatchley *et al*. [83] on the sensitivity of microorganisms to UV-C radiation, including SARS-CoV-2, both providing the current state of the field for inactivation of microorganisms and UV-C radiation dose.

### 6.3 International Conference on UV Disinfection for Air and Surfaces, December 8–9, 2020

NIST partnered with IUVA on its International Conference on UV Disinfection for Air and Surfaces. This first-time event was a successful demonstration of the continued engagement of industry leaders involved in all aspects of air and surface UV-C disinfection innovation and device development. The conference addressed key validation aspects of optics, radiometry, and photobiology, as well as the latest developments in UV-LEDs and far UV-C, with these topics included in the paper by Spicer [44]. For a review of far UV-C (200 nm to 230 nm) radiation, see Ref. [47].

### 6.4 Public Webinar on “Enhancing the New Normalcy with UV Disinfection,” April 27 and 29, 2021

NIST partnered with IUVA for the public webinar “Enhancing the New Normalcy with UV Disinfection.” By early 2021, it was clear the SARS-CoV-2 pandemic had delivered a profound and negative impact to communities worldwide, with the suspension of normal activities and lasting effects on all people and societies. UV-C technology has been continuously looked upon as one that can help with a return to normalcy. This event established a forum to encourage dialogue among stakeholders involved in all aspects of UV-C disinfection to consider how UV-C technology can contribute to measures and policies for resuming normalcy in society. Potential UV-C disinfection strategies, concerns, and regulatory barriers in restarting normal activities were addressed, with an emphasis on UV-C technology basics, followed by topical panels addressing key aspects of UV-C use in public, commercial, and residential buildings and transportation sectors. See the paper by Yates *et al*. [84] for the latest understanding, and a fundamental review, of UV-C radiation interactions with materials in commercial aircraft.

Speakers at the event also discussed commercial and consumer applications targeting all aspects of the COVID-19 pandemic, leading to a closing webinar panel that examined current policy and guidance for the use of UV-C disinfection in diverse settings and applications. The closing webinar panel concluded that peer-reviewed research publications are essential to help support the development of guidance, standards, and regulations for UV disinfection technologies.

## 7 Looking Ahead: Standards, Innovation Ecosystems, and Technology Transfer

### 7.1 Standards

The main objective for all stakeholders engaged in the development and implementation of UV disinfection technologies is to advance standards and data requirements for the technology to be more uniformly applied to healthcare for health, innovation, and security. As described by Poster *et al*. [58], collaborative partnerships will be essential to meet this objective. These collaborations are ongoing through different types of engagements and partnerships on workshops, conferences, and webinars (Table 2). Through these efforts and the IUVA healthcare WG (see Sec. 4.3), a UV standards roadmap plan has been developed (Table 6) to help meet the needs identified at the NIST–IUVA workshop (Fig. 8). With respect to antimicrobial efficacy and dose-response curves, achievement of specified log reductions in pathogens on carriers at specified distances and exposures is of great importance, with alternative approaches such as fluence being considered as a measurand of equal importance.

Results from the papers in this special section of the *Journal of the National Institute of Standards and Technology* (Table 1) will provide useful information towards completing the UV standards roadmap plan and meeting the needs of the future. For example, to the latter point, Obeng *et al*. [85] correlated fluence to electrical properties of thin films of bacteriophage lambda double-stranded DNA and demonstrated how new analytical techniques can be applied to measure log reductions of biological inactivation. The IUVA healthcare WG will continue to coordinate UV standards development through its efforts with the UV community and its public and private sector stakeholders and support development of UV for health, UV for innovation, and UV for security, as was identified at the NIST–IUVA workshop (Fig. 8).

**Table 6:** UV Standards Roadmap Plan—A Proposed Scope^a^ [58]

Name	Plans
1. Standard for measuring UV lamp and luminaire irradiance	1.1. Gaseous discharge lamps (Hg, xenon, continuous and pulsed) 1.2. LEDs (continuous and pulsed) 1.3. Excimers and lasers
2. Standard for measuring UV luminaire antimicrobial efficacy	2.1. Surface (two-dimensional and three-dimensional; *e.g.*, whole-room devices, cabinet enclosures, handheld devices, mobile devices) 2.2. Air (*e.g.*, heating, ventilation, and air conditioning, internal, upper air, and portable air-filter UV devices) 2.3. Water (*e.g.*, utility system treatment such as potable water or municipal wastewater, building system treatment, including commercial and residential, agriculture, and point of use)
3. Standards for calibrating UV irradiance measurement systems	3.1. Radiometers—intensity of radiant energy 3.2. Dosimeters—industrial and personal needs 3.3. Other
4. Standards for measuring UV transmittance and absorption	4.1. Gaseous—air applications 4.2. Liquid—water applications 4.3. Solid—surfaces, matter interaction applications
5. Standards for measuring UV dose response curves and action spectra	5.1. Pathogen specific, for specific wavelengths (*e.g.*, 222 nm, 254 nm, 265 nm, 405 nm) 5.2. Pathogen specific across the continuous UV spectrum (from 150 nm to 400 nm or more)

^a^ Developed by the IUVA healthcare WG with input from internationally engaged organizations such as the American Society of Heating, Refrigerating and Air-Conditioning Engineers (ASHRAE); International Commission on Illumination (CIE); International Organization for Standardization (ISO); ASTM International; Association for the Advancement of Medical Instrumentation (AAMI); and Underwriters Laboratories (UL).

The UV standards roadmap plan in Table 6 also includes standards for calibrating UV irradiance measurement systems. Calibrations are uniquely supported by NIST’s photometry laboratory and its specialized radiation source facilities [86]. The laboratory is helping to meet the industry’s UV calibration needs, especially as demands are increasing and changing. For example, a recent update to the laboratory provides the NIST LED brightness and photometer calibration service with measurement uncertainties of 0.2% or less. Plans are under way for adding a goniophotometer to offer a new type of service: measuring LED UV output. This is important because recent research suggests that UV LEDs with peak emission at approximately 286 nm could serve as an effective tool in the fight against human coronaviruses [87]. NIST’s photometry laboratory is an example of a component within an innovation ecosystem [88] that enables services to support innovation by coupling the “resources available to the knowledge economy with resources generated by the commercial economy” [89].

### 7.2 Innovation Ecosystems

The large group of different types of stakeholders engaged in the collaborative efforts summarized in Tables 1 and 2 and detailed in Secs. 4–6 reflects a range of entities that is a necessary component within an innovation ecosystem [88]. These entities include those involved in research collaborations (*i.e.*, public-private partnership members, foundation members, and consortia members) [88]. Federal assets help to inject scientist-support, technologies, and research capacities, such as user facilities like the NIST photometry laboratory [86], and tie these capacities to the research collaborations and other assets such as incubators, accelerators, investors, economic development organizations, and federal funding arrangements [88].

Poster *et al.* [58] reviewed many collaborative models, including the government, in the context of the UV industry and provided an example consortium for enabling the sharing of precompetitive research to support UV standards development for healthcare. Innovative ecosystems are a possible model that can incorporate the proposed consortium to better enable “the translation of scientific research outcomes from the public and private sectors into products and services that can be used by the public” [88], such as uniformly characterized, adopted, and implemented UV-C devices for the disinfection of a whole-room environment in a healthcare facility.

A UV industry innovation ecosystem, with the incorporation of a consortium component, “may lead to new scientific truths and reliable methods for knowledge and economic development” [90]. Such a development could support all three of the critical areas identified by UV stakeholders at the NIST–IUVA workshop: UV for health, UV for innovation, and UV for security (Fig. 8).

### 7.3 Technology Transfer

There is a growing interest in the public value of technology transfer and technology transfer mechanisms. The criterion arises from the recognition that the public sector transfer of technology comes from agencies and organizations that pursue public-interest goals, such as better public health, through their missions [91]. Collaborative efforts among NIST, IUVA, and other partners are an example of technology transfer mechanisms that are driven by public value. For UV disinfection technologies to transfer to the good of the public, reciprocal relationships between entities (*i.e.*, government, industry, and academia partnerships) must occur [91]. Alternatively, open-source approaches that enable knowledge transfer catalyze standards creation and further knowledge sharing among collaborators in an ecosystem. In this approach, technology transfer happens at an accelerated rate due to the standards development and knowledge sharing cycling within the ecosystem [91]. Both partnerships and open-source approaches were reviewed by Poster *et al.* [58] in the context of the collaboration between NIST, IUVA, and others.

Interestingly, the publications in Table 1, which are outputs from the sequence of events in Table 2, are a common approach used to define the needs for standard setting in an industry. Poster *et al*. [58] reviewed similar approaches used for the emerging semiconductor industry, and more recent examples include the nanotechnology and photopolymer additive manufacturing industries [92], all cases where communities connected first through workshops to define and set agendas for research needs, priorities, or grand challenges that could only be overcome through the development of standards and culminate with transfer of technologies for the benefit of the public. Bozeman *et al*. [91] noted that such events lead to public policies through stages, with agenda setting (*i.e.*, workshops) being the first step.

Success metrics for technology vary, and qualitative measures of public value are one approach. The main question to assess the public value criterion is, Did technology transfer enhance the collective good and meet broad, societally shared values? [91]. We believe the transfer of ideas from the events described in this paper, and the papers in this special section of the *Journal of Research of the National Institute of Standards and Technology*, accomplished this for better public health and security through economic development. All these values are important to UV stakeholders and tied to the critical areas identified at the NIST–IUVA workshop: UV for health, UV for innovation, and UV for security (*i.e.*, economic development and growth of economies of scale) (Fig. 8).

Lasty, Anderson and Breitzman [93] reviewed technology transfer impacts due to NIST engagement in innovative ecosystems. The impact was measured through outputs, such as workshops, presentations publications, and standards. Based on Table 1 (publications) and Table 2 (workshops, presentations), success metrics for meeting public value benefits through technology transfer mechanisms are being met in the UV industry. A future assessment metric could be the downstream impacts from the publications and subsequent publications and the influence of these on patents [94]. Patents are fueled and sustained through innovation. Innovation is one of the critical areas identified at the NIST–IUVA workshop (*i.e.*, UV for innovation), and it ties directly into the other critical areas identified at the workshop (*i.e.*, UV for health and UV for security) (Fig. 8). The research directions identified at the NIST–IUVA workshop (Fig. 8) are the logical next steps required to meet public value benefits for UV technologies in healthcare through standards, innovative ecosystems, and technology transfer mechanisms.

## Supplementary Material

Supplementary material is provided with supporting information from the many NIST–IUVA collaborative engagements from 2017 to 2021. Supplemental figures and tables provide information, such as photos, agendas, participants, and speakers from the events. Most notably, all abstracts and presentations (talks and posters) from the NIST-IUVA Workshop on Ultraviolet Disinfection Technologies that took place in Gaithersburg, Maryland, USA, January 15–16, 2020, are available through URL links in Supplemental Table 5. Descriptions of the collaborative engagements are provided in the article text in Sec. 4 (NIST and Community Engagement for the Development of UV Efficacy Standards), Sec. 5 (NIST–IUVA Workshop on UV Technologies for Healthcare and Metrology Needs) and Sec. 6 (Outcomes of the Workshop). A full description of each element in the supplementary material is given in Supplemental Table 1.

Supplementary material is available at https://doi.org/10.6028/jres.126.014s.
